# Radiomic Analysis of Treatment Effect for Patients with Radiation Necrosis Treated with Pentoxifylline and Vitamin E

**DOI:** 10.3390/tomography10090110

**Published:** 2024-09-09

**Authors:** Jimmy S. Patel, Elahheh Salari, Xuxin Chen, Jeffrey Switchenko, Bree R. Eaton, Jim Zhong, Xiaofeng Yang, Hui-Kuo G. Shu, Lisa J. Sudmeier

**Affiliations:** Department of Radiation Oncology, Winship Cancer Institute of Emory University, Atlanta, GA 30322, USA

**Keywords:** radiation, necrosis, pentoxifylline, vitamin E, radiomics

## Abstract

**Simple Summary:**

Patients who undergo stereotactic radiosurgery (SRS) are at risk of developing radiation necrosis (RN), which may lead to permanent neurological injury. While corticosteroids are common in first-line treatment, they carry their own side effect profile. Prior studies have shown that the combination of pentoxifylline (Ptx) and vitamin E (VitE) may be a possible alternative management route for RN. In this study, we provide our institutional experience on the use of Ptx + VitE in patients who developed RN after SRS. We found that nearly half of our patients showed some evidence of improvement on MRI with Ptx + VitE, with very few side effects. Furthermore, deeper analyses of post-contrast MRIs show that derivable radiomic features may predict how patients will respond to this treatment. Our results suggest that Ptx + VitE for RN and radiomic analysis of treatment response be evaluated in a larger-scale study.

**Abstract:**

Background: The combination of oral pentoxifylline (Ptx) and vitamin E (VitE) has been used to treat radiation-induced fibrosis and soft tissue injury. Here, we review outcomes and perform a radiomic analysis of treatment effects in patients prescribed Ptx + VitE at our institution for the treatment of radiation necrosis (RN). Methods: A total of 48 patients treated with stereotactic radiosurgery (SRS) had evidence of RN and had MRI before and after starting Ptx + VitE. The radiation oncologist’s impression of the imaging in the electronic medical record was used to score response to treatment. Support Vector Machine (SVM) was used to train a model of radiomics features derived from radiation necrosis on pre- and 1st post-treatment T1 post-contrast MRIs that can classify the ultimate response to treatment with Ptx + VitE. Results: A total of 43.8% of patients showed evidence of improvement, 18.8% showed no change, and 25% showed worsening RN upon imaging after starting Ptx + VitE. The median time-to-response assessment was 3.17 months. Nine patients progressed significantly and required Bevacizumab, hyperbaric oxygen therapy, or surgery. Patients who had multiple lesions treated with SRS were less likely to show improvement (*p* = 0.037). A total of 34 patients were also prescribed dexamethasone, either before (7), with (16), or after starting (11) treatment. The use of dexamethasone was not associated with an improved response to Ptx + VitE (*p* = 0.471). Three patients stopped treatment due to side effects. Finally, we were able to develop a machine learning (SVM) model of radiomic features derived from pre- and 1st post-treatment MRIs that was able to predict the ultimate treatment response to Ptx + VitE with receiver operating characteristic (ROC) area under curve (AUC) of 0.69. Conclusions: Ptx + VitE appears safe for the treatment of RN, but randomized data are needed to assess efficacy and validate radiomic models, which may assist with prognostication.

## 1. Introduction

Stereotactic radiosurgery (SRS) is a common treatment for brain metastases, benign brain tumors such as meningiomas, and arteriovenous malformations (AVMs) that are not amenable to surgery. SRS is non-invasive and confers excellent rates of local control with minimal side effects in most patients. In approximately 5–10% of patients, however, SRS causes radiation necrosis (RN), a condition characterized by imaging changes with or without associated neurologic symptoms [1,2]. RN can be difficult to distinguish from tumor recurrence or tumor pseudo-progression. MRI changes can include increased edema on T2 and increased contrast enhancement from alterations to the blood–brain barrier [3]. The risk of developing RN varies with factors such as dose/fractionation of radiation, initial vs. re-treatment, and concurrent or subsequent administration of systemic agents [4]. Neurologic symptoms associated with RN can include headaches, nausea, and vision changes and may mimic the symptoms associated with the initial presentation of the treated lesion itself, such as focal motor or sensory deficits. The natural history of RN varies across patients. In some, symptoms never develop despite observed changes in imaging. In others, it causes devastating progressive neurologic decline [1].

The pathophysiology underlying RN has been investigated but is still not clear. One explanation focuses on the breakdown of the blood–brain barrier due to endothelial cell dysfunction, which causes cerebral edema [5]. Occlusive disease in small cerebral vessels is thought to cause necrosis of glial cells, leading to loss of myelin [1]. Chronic inflammation is also thought to play a role, and there is evidence that vascular endothelial growth factor (VEGF) may be involved in promoting capillary permeability in RN [6,7,8].

Corticosteroids are a common initial treatment for symptomatic RN with the goal of reducing cerebral edema. While some patients do improve with a short course of corticosteroids, others require long-term medical management of RN. Although steroids often achieve symptomatic improvement, the side effects of sustained use may include, but are not limited to, sleep disturbances, elevated blood glucose, gastrointestinal symptoms, anxiety, osteoporosis, and a rare risk of avascular necrosis of the hip, which limits its long-term use [9]. Consistent with the role of VEGF in the pathophysiology of RN, bevacizumab is recommended when steroids are not effective or when symptoms return after tapering off a long course of steroids. Bevacizumab may provide symptomatic relief, radiographic improvement, and reduced need for steroids [10]. Anticoagulants such as warfarin and heparin have also been investigated, with limited benefit and an increased risk of bleeding [11].

Alpha-tocopherol or vitamin E (VitE) is one of the fat-soluble vitamins and an important antioxidant that facilitates the protection and maintenance of cell membranes and, transitively, endothelial integrity. Pentoxifylline (Ptx) acts against inflammatory mediators and decreases the viscosity of blood to facilitate blood flow through small-caliber or damaged vessels. The combination of oral Ptx and VitE, which has been used to treat radiation-induced fibrosis and soft tissue injury, has also been investigated as a treatment for RN [12,13]. In 2008, Williamson et al. showed an average decrease in edema with the use of Ptx + VitE in 11 patients with suspected radiation necrosis after SRS [14]. To our knowledge, there have been no other published reports studying the efficacy of Ptx + VitE for the treatment of RN. At our institution, we treat patients with symptomatic RN as well as those with radiographic evidence of RN without symptoms. Treatment regimens include dexamethasone with or without Ptx + VitE or a trial of Ptx + VitE alone or after a patient no longer tolerates the side effects of dexamethasone. Here, we present a single institutional observational data on outcomes among patients prescribed Ptx + VitE at Emory University for the treatment of RN and how radiomics may potentially assist in the assessment of treatment response.

## 2. Materials and Methods

### 2.1. Patients

The Emory Department of Radiation Oncology database was retrospectively searched to identify patients who had undergone radiation therapy for any diagnosis and who subsequently were treated with Ptx + VitE from January 2012 to June 2018. Patients were prescribed 400 mg of Ptx twice a day + 1000 IU of VitE daily. The electronic medical record of identified patients was reviewed to select patients with a diagnosis of RN who had magnetic resonance imaging (MRI) before and after starting Ptx + VitE. The diagnosis of RN was made by the radiation oncologists based on patient symptoms and/or imaging features, including enlargement of the contrast-enhancing volume with low suspicion for tumor progression.

### 2.2. Response Assessment

The radiation oncologists recorded their impression of the imaging from before and after starting Ptx + VitE and the patient’s symptoms (if present) in the electronic medical record at each patient visit. This impression was used to score each patient’s response to treatment using the following six categories: no change, improvement, worsening, mixed features of improvement and worsening, disease progression, worsening RN with disease progression.

### 2.3. Statistical Analysis

Descriptive statistics were generated for categorical variables using frequencies and percentages and for numeric variables using mean, median, standard deviation, and range. Improvement and worsening based on the radiation oncologist’s impression of the post-Ptx + VitE MRI read were compared across patient characteristics using chi-squared tests, Fisher’s exact tests, or ANOVA where appropriate. Statistical analysis was performed using SAS 9.4 (SAS Institute Inc., Cary, NC, USA), and statistical significance was assessed at the 0.05 level.

### 2.4. Radiomics

#### 2.4.1. Clinical Data Collection

A total of 43 patients (who had follow-up magnetic resonance images (MRI) at least 1 month after Ptx + VitE) that could be classified as stable, improved, or worsened on treatment were selected for this analysis. The 5 patients with mixed response or disease progression were excluded from this analysis. These 43 patients were divided into three classes as follows: class 0 = stable disease (n = 9); class 1 = improvement (n = 21); class 2 = worsening (n = 14, includes 1 with mixed response and 1 with RN + tumor progression).

#### 2.4.2. Pre-Processing

The post-contrast T1-weighted MRI sets for each patient were segmented slice-by-slice in the axial plane using Velocity software (Ver. 4.1 Varian Medical System Inc, Palo Alto, CA, USA). These segmented sets were then transferred to the open-source 3D slicer software (Ver 5.0.3) for feature extraction [15,16]. Prior to feature extraction, wavelet filters (high-pass (H) and low-pass (L)) were applied to the 3D images, resulting in eight unique filter combinations (HHH, HHL, HLH, LHH, LLL, LLH, LHL, HLL) [17].

#### 2.4.3. Feature Extraction

Radiomics features including 19 first-order statistics, 10 2D shape-based, 16 3D shape-based, 24 Gray Level Co-occurrence Matrix (GLCM), 16 Gray Level Run Length Matrix (GLRLM), 16 Gray Level Size Zone Matrix (GLSZM), 5 Neighboring Gray Tone Difference Matrix (NGTDM), and 14 Gray Level Dependence Matrix features (GLDM) were extracted from T1-post-contrast MRI using Pyradiomics (Ver 3.0.1) [18]. In total, 120 features were computed and extracted from the original and preprocessed (with each of the 8 wavelet filter sets) images, resulting in 1080 radiomics features from the pre- and post-therapy T1-post-contrast weighted region of interest (ROI) for each patient. A Linear Discriminant Analysis (LDA) was applied for feature dimension reduction [19]. Final analysis utilized only radiomic features from the post-therapy scans because features from the pre-therapy scan did not further increase the predictive power of the classification models that were developed below.

#### 2.4.4. Classification

First, standardization was implemented on feature values to rescale data onto a range between zero and one using MinMaxScaler [20]. Due to the small sample size, Support Vector Machine (SVM) was trained for classification [21]. To account for our small sample size and to address any dataset imbalances that may cause overfitting, we applied synthetic minority over-sampling technique (SMOTE) [22]. A Nested Leave-One-Out Cross-Validation was performed to evaluate the classification performance and tune hyperparameters [23]. Finally, the model performance was assessed by the receiver operating characteristic (ROC) curve with the area under curve (AUC) as the primary metric [24].

## 3. Results

### 3.1. Descriptive Data

We identified 48 patients who developed RN after radiation therapy and were treated with Ptx + VitE [25]. The majority of these patients (45) were treated with SRS in 1–5 fractions. Only three patients with atypical meningiomas were treated with conventionally fractionated radiation therapy, each receiving 59.4 Gy in 33 fractions of 1.8 Gy each (Table 1). Over half of the patients (25) were treated for brain metastases (13—NSCLC, 6—Melanoma, 2—Breast Cancer, 4—Other, including Ovarian, Renal Cell Carcinoma, small-cell lung cancer, small-cell carcinoma of unknown origin). Arteriovenous malformations (AVMs) were treated in 13 patients; meningiomas were treated in 8 patients, and treatment was performed for 1 patient each with hemangioblastoma and glomus jugulare tumor. The most common location for a RN lesion was in the frontal lobe (19 of 48 patients), and most patients in this cohort developed RN in a lesion that had only been irradiated once (85.5%). A total of 17 patients (35.4%) had multiple other separate lesions treated with radiation therapy as well (Table 2).

Less than half (43.8%) of the patients in this cohort had asymptomatic RN and were treated on the basis of imaging findings alone. On the other hand, the most common RN-associated symptoms included seizures (eight patients), impaired co-ordination (five patients), headache (four patients), and weakness (four patients). Other reported symptoms were vision changes (two patients), confusion (two patients), facial droop (one patient), and speech changes (one patient). The median time from completion of radiation to MRI findings of RN was 1.26 years (range: 0.25–10.2 years; Table 2).

### 3.2. Use of Ptx + VitE with Other RN Treatments

In 25 patients (52.1%), Ptx + VitE was started as the initial RN treatment. Of that cohort, 12 required no other RN treatment, 11 subsequently required treatment with dexamethasone, 1 patient went on to receive hyperbaric oxygen therapy, and 1 had tumor progression requiring treatment with SRS (Table 3). In the other 23 patients, Ptx + VitE was started at the same time as dexamethasone in 16 cases (33.3%) and, in 7 cases (14.6%), after dexamethasone was initiated. Overall, 10 patients required additional non-steroid RN interventions beyond Ptx + VitE, including 3 patients who received hyperbaric oxygen therapy, 3 patients who received bevacizumab, 3 patient who underwent surgery, and 1 who was treated with SRS for tumor progression (Table 3).

### 3.3. Side Effects Associated with Ptx + VitE

Three patients stopped Ptx + VitE early due to reported side effects (Table 3); two of whom were among the twelve patients who required no other RN treatment beyond Ptx + VitE. One patient reported leg cramps and headaches and stopped treatment after 3 weeks. The other reported nausea and stated that they stopped the medication soon after starting. The third patient who stopped treatment due to side effects was the subject who required hyperbaric oxygen therapy after a trial of Ptx + VitE monotherapy. This patient reported discontinuing treatment after a few weeks due to nausea. These three patients were the only ones who reported side effects associated with Ptx + VitE treatment; no patients who were on both dexamethasone and Ptx + VitE reported side effects from the treatment.

### 3.4. Assessment of Imaging Response after Initiating Ptx + VitE

The median time-to-response assessment with MRI after starting Ptx + VitE was 3.17 months (range: 0.66–12.68 months; Table 4). Among the entire cohort of patients, 30 (62.5%) showed RN that was either stable or improved (Table 4). To better assess the impact of Ptx + VitE in isolation from other treatments, we determined the response among the 12 patients who were treated with Ptx + VitE alone. In this cohort, RN was stable or improved in nine (75%, five showing improvement and four being stable), with only three showing progression based on the radiation oncologist’s impression of the follow-up MRIs after starting Ptx + VitE. One of two patients in the Ptx + VitE monotherapy cohort who stopped treatment early due to side effects had progressive RN upon follow-up imaging. Of the tested variables, only the presence of multiple irradiated lesions was significant for interaction with response to Ptx + VitE, with these patients being less likely to show improvement upon imaging after Ptx + VitE, *p* = 0.037 (Table 5). A multivariable logistic regression model that included the variables with the lowest *p* values from the ANOVA (Other lesions treated, Diagnosis, and Dexamethasone) showed an odds ratio for improvement on Ptx + VitE of 0.28 for having multiple lesions treated (CI 0.07–1.08, *p*-value 0.064, Table 6).

### 3.5. Radiomics Assessment of Treatment Response

**Figure 1:**
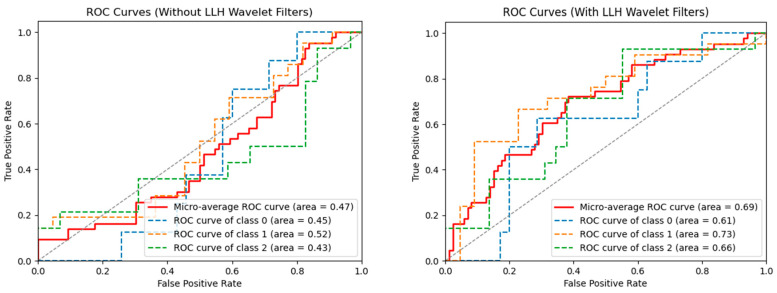
ROC or machine learning models without LLH wavelet filters (**left**) and with LLH wavelet filters (**right**). Both models have the SMOTE technique applied to correct for dataset imbalances. As seen on the right, LLH with SMOTE had the best performance for all classes of patients: class 0 = stable disease (n = 9); class 1 = improvement (n = 21); class 2 = worsening (n = 14).

SVM machine learning models with and without wavelet filters were developed and further corrected for imbalance and overfitting with and without SMOTE. While the model developed using the original, unprocessed images with SMOTE was not predictive, with an average AUC of 0.50, the optimal model using an LLH pre-processing filter with SMOTE gave an average AUC of 0.69 (Figure 1). The most significant radiomics features for these models are available in Supplementary Table S1. The performance of models utilizing the full combinations of wavelet filters and SMOTE is also shown in Supplementary Table S2.

## 4. Discussion

This retrospective institutional study aimed to delineate the utility of Ptx + VitE for the management of radiation necrosis for patients treated with CNS-related pathologies. The majority of the patients in our cohort were treated with SRS versus conventionally fractionated radiation therapy. Regardless of modality of treatment, radiation necrosis is a late side effect of radiation therapy, believed to involve a complex interaction between the vasculature and connective tissue, leading to a triad of ischemia, fibrosis, and necrosis. A recent study by Kerschbaumer et al. evaluated potential risk factors for RN with SRS and found that for patients treated for brain metastases, Cox regression showed an increased hazard ratio for patients with large diameter disease or treated with a high dose (HR 1.065, *p* = 0.028 and HR 1.302, *p* < 0.001, respectively) [26]. In our own institution, a dosimetric analysis of five-fraction SRS to surgically resected metastatic brain lesions showed that a high maximum dose and hot spots within the PTV margin may predict radiation necrosis [27].

In the clinic, RN is usually diagnosed radiographically, although the appearance of RN versus tumor recurrence has significant overlap on standard MRIs. Consequently, additional methods to help distinguish the progression of disease from RN have been investigated with several studies utilizing radiomics to make this distinction [28,29,30]. Despite advances in this area, there continue to be instances in which surgical resection of presumed progression returns as RN or vice versa.

In general, the first line of treatment for RN is usually steroids with alternatives, including bevacizumab or hyperbaric oxygen. Bevacizumab is a monoclonal antibody that inhibits vascular endothelial growth factor. Systematic reviews on the use of bevacizumab for RN from brain metastasis showed some efficacy; however, the level of evidence supporting its use was low, and questions such as timing of medication and dose remained unanswered [31,32]. In a similar vein, hyperbaric oxygen addresses the angiogenesis pathway by promoting oxygenation. While seen to have benefits in several case reports, the recommendation of hyperbaric oxygen for RN is hampered by limited prospective evidence, access, and costs, as well as associated side effects [33,34].

An additional approach to managing RN is to use Ptx and VitE. One of the earliest experimental studies by Lefaix et al. showed that the combination of Ptx and VitE helped decrease fibrosis in pig models that were treated with gamma rays [35]. More recent studies have shown a decrease in fibrosis in breast tissue as well as in the management of osteoradionecrosis of the mandible [12,36,37]. Williamson et al. showed that for patients with RN, the use of Ptx and VitE decreased the mean volume of edema in their 11-patient cohort, with the only adverse effects being nausea and abdominal discomfort in two patients [14]. Within this cohort of 48 patients whose treatment paradigm for RN involved Ptx + VitE, 30 (62.5%) patients either showed improvement or stable disease on follow-up MRI after Ptx + VitE; only 3 patients (6.25%) reported side effects from this therapy. Similar to Williamson’s work, these side effects primarily consisted of nausea.

Nearly 50% of our patients who started Ptx + VitE monotherapy (12 of 25 patients) required no other intervention. Moreover, the use of Ptx + VitE did not add toxicity to dexamethasone; no patients on Ptx + VitE with dexamethasone reported side effects from treatment. It is possible, however, that side effects from dexamethasone may have masked any side effects from Ptx + VitE. A total of 75% of patients who were treated with Ptx + VitE alone (9 of 12) had either stable or improved RN on imaging. It is unknown how these nine patients would have fared without Ptx + VitE. Randomized data are needed to definitively determine whether Ptx + VitE monotherapy is effective or whether its addition to dexamethasone confers any benefit. In addition, caution must be advised when prescribing to patients with cardiovascular disease as Ptx is a vasoactive agent.

Only 3 of the 12 patients who were treated with Ptx + VitE alone had symptomatic RN. One of the three symptomatic patients had progression of RN on imaging, and the other two had stable RN after treatment with Ptx + VitE. Since most of the cohort that received Ptx + VitE monotherapy was asymptomatic, our data mostly support the use of Ptx + VitE monotherapy in cases of asymptomatic RN.

Historically, MRI has been used for visual assessment of tumor response to therapy [38]. Unfortunately, this method has drawbacks, such as the experience of the reading radiologist, machine calibration, patient movement, and time-dependent anatomical changes, resulting in variability amongst final reads [39]. Furthermore, pre-treatment and early post-treatment MRIs could have subtle features/changes that may not be appreciated by normal visual assessment. In our study, we sought to improve on typical visual evaluation by assessing radiomics features that were extracted from T1 post-contrast MRIs. We felt that this approach of utilizing a range of quantitative imaging features extractable from the relevant scans might provide more objective prognostic information on the response of RN to the Ptx + VitE. Based on the limited number of patients within the training set, we felt that the most appropriate approach would be to utilize the “leave-one-out” training method. With this, our best model using the LLH wavelet filters for the preprocessing of image sets with SMOTE resulted in ROC curves that achieved an overall AUC of 0.69 ± 0.091. Our results align with other studies that demonstrate the effectiveness of preprocessing filters in improving machine learning model performance [40,41]. While limited in the number of patients used to develop this model, these data show that it is feasible to use a radiomics-based approach to evaluate RN treatment response and represent groundwork for larger validation studies. Future prospective studies would be useful in training our model to enhance sensitivity and specificity with the goal of prognosticating outcomes of patients with RN early in their therapeutic course.

## 5. Conclusions

RN is a rare, late side effect of radiation therapy that necessitates management. While first-line steroids are well known, their side effect profile can be limiting in some patients. Other alternatives, such as bevacizumab and hyperbaric oxygen, are also limited based on accessibility, cost, and lack of prospective data. In this institutional study, we corroborate previous findings of the utility of Ptx + VitE for late side effects of radiation therapy and offer one of the largest CNS-based cohorts within whom Ptx + VitE appears to be a safe and accessible alternative for RN management. Finally, quantitative radiomics features extractable from pre- and early post-treatment MRIs provide additional prognostic information on the response to Ptx + VitE. Future work, including randomized assessments, will be vital to determine efficacy and comparability between treatment options for RN as well as to validate and further refine radiomic analyses as a prognosticator of response.

## Figures and Tables

**Table 1:** Radiation details.

Variable		N = 48
Dose/fraction (Gy)	Mean/Median	14.00/17.50
	Range	1.80–21
	Std Dev	5.94
	Missing	1
Number of fractions	Mean/Median	3.88/1
	Range	1–33
	Std Dev	7.76
	Missing	0

**Table 2:** Descriptive data.

Variable		N = 48	%
Sex	Male	24	50
	Female	24	50
Age at treatment	Average	55 y	
	Interquartile range	20.2 y	
Diagnosis	Meningioma	8	16.7
	Non-small-cell lung cancer (NSCLC)	13	27.1
	Arteriovenous Malformation (AVM)	13	27.1
	Breast cancer	2	4.2
	Melanoma	6	12.5
	Other	6	12.5
Location of lesion	Frontal	19	39.6
	Parietal	9	18.8
	Temporal	4	8.3
	Occipital	4	8.3
	Posterior fossa	7	14.6
	Thalamic	1	2.1
	Other	4	8.4
Was the RN lesion re-irradiated?	Yes	7	14.6
	No	41	85.4
Were other lesions treated?	Yes	17	35.4
	No	31	64.6
RN-associated symptoms pre-Ptx + VitE	Asymptomatic	21	43.8
	Headache	4	8.3
	Seizures	8	16.7
	Weakness	4	8.3
	Impaired co-ordination	5	10.4
	Vision	2	4.2
	Facial droop	1	2.1
	Confusion	2	4.2
	Speech	1	2.1
Interval from treatment completion to RN presentation (years)	Mean	1.92	-
	Median	1.26	-
	Minimum	0.25	-
	Maximum	10.22	-
	Std Dev	1.85	-

**Table 3:** Therapies utilized and adherence to Ptx + VitE.

Variable		N = 48	%
Dexamethasone	None	14	29.2
	Started w/Ptx + VitE	16	33.3
	Started after Ptx + VitE	11	22.9
	Started before Ptx + VitE	7	14.6
Non-steroid therapies used in addition to Ptx + VitE	None	38	79.2
	Bevacizumab	3	6.3
	Hyperbaric oxygen	3	6.3
	Surgery	3	6.3
	SRS	1	2.1
Reported adherence to Ptx + VitE Prescription	Yes	43	89.6
	Stopped early due to side effects	3	6.3
	Unclear	2	4.2

**Table 4:** Imaging response to Ptx + VitE.

Variable		N = 48	%
Radiation oncologist’s assessment of MRI after Ptx + VitE	No change	9	18.8
	Improvement	21	43.8
	Worsening	12	25.0
	Mixed	1	2.1
	Disease progression	4	8.3
	Worsening RN + disease progression	1	2.1
Interval from starting Ptx + VitE to MRI assessment (Months)	Mean	4.52	-
	Median	3.17	-
	Minimum	0.66	-
	Maximum	12.68	-
	Std Dev	2.64	-
	Missing	0	-

**Table 5:** Comparison between selected variables and improvement of RN on MRI after Ptx + VitE.

			Improved on Ptx + VitE		
Covariate	Statistics	Level	No N = 27	Yes N = 21	*p*-Value *
Diagnosis	N (Col %)	Meningioma/NSCLC	14 (51.85)	7 (33.33)	0.199
	N (Col %)	Other	13 (48.15)	14 (66.67)	
Location	N (Col %)	Frontal	10 (37.04)	9 (42.86)	0.683
	N (Col %)	Other	17 (62.96)	12 (57.14)	
Re-treatment	N (Col %)	Yes	4 (14.81)	3 (14.29)	1.000
	N (Col %)	No	23 (85.19)	18 (85.71)	
Other lesions treated	N (Col %)	Yes	13 (48.15)	4 (19.05)	**0.037**
	N (Col %)	No	14 (51.85)	17 (80.95)	
Symptoms pre pentoxi	N (Col %)	Yes	14 (51.85)	13 (61.9)	0.486
	N (Col %)	No	13 (48.15)	8 (38.1)	
Dexamethasone	N (Col %)	Yes	18 (66.67)	16 (76.19)	0.471
	N (Col %)	No	9 (33.33)	5 (23.81)	
Years from treatment to post-MRI	N		27	21	0.964
	Mean		2.24	2.26	
	Median		1.99	1.55	
	Min		0.44	0.45	
	Max		5.69	10.74	
	Std Dev		1.48	2.37	
Months from pre-MRI to post-MRI	N		27	21	0.296
	Mean		4.17	4.98	
	Median		3.29	3.06	
	Min		1.15	0.66	
	Max		12.68	11.01	
	Std Dev		2.49	2.81	

* The *p*-value is calculated by ANOVA for numerical covariates and chi-square test or Fisher’s exact for categorical covariates, where appropriate.

**Table 6:** Post-MRI improvement, multivariate analysis *.

		Improved on Ptx = Yes	
Covariate	Level	Odds Ratio (95% CI)	OR *p*-Value
Other lesions treated	Yes	0.28 (0.07–1.08)	0.064
	No	-	-

Diagnosis	Other	1.93 (0.56–6.63)	0.297
	Meningioma/NSCLC	-	-

Dexamethasone	Yes	0.80 (0.20–3.20)	0.752
	No	-	-

* Number of observations in the original data set = 48. Number of observations used = 48.

## Data Availability

The data that support the findings of this study are available from the corresponding author upon reasonable request.

## Supplementary Materials

The following supporting information can be downloaded at: https://www.mdpi.com/article/10.3390/tomography10090110/s1, Table S1: Highest weighted radiomic features in SVM models of original and LLH filtered images; Table S2: Performance of radiomics models with varying wavelet filters with and without SMOTE.

## References

[1] Giglio P., Gilbert M.R. (2003). Cerebral radiation necrosis. Neurologist.

[2] Ruben J.D., Dally M., Bailey M., Smith R., McLean C.A., Fedele P. (2006). Cerebral radiation necrosis: Incidence, outcomes, and risk factors with emphasis on radiation parameters and chemotherapy. Int. J. Radiat. Oncol. Biol. Phys..

[3] Miyatake S.-I., Nonoguchi N., Furuse M., Yoritsune E., Miyata T., Kawabata S., Kuroiwa T. (2015). Pathophysiology, Diagnosis, and Treatment of Radiation Necrosis in the Brain. Neurol. Med.-Chir..

[4] Marks J.E., Bagĺan R.J., Prassad S.C., Blank W.F. (1981). Cerebral radionecrosis: Incidence and risk in relation to dose, time, fractionation and volume. Int. J. Radiat. Oncol. Biol. Phys..

[5] Cheng K.-M., Chan C.-M., Fu Y.-T., Ho L.-C., Cheung F.-C., Law C.-K. (2001). Acute hemorrhage in late radiation necrosis of the temporal lobe: Report of five cases and review of the literature. J. Neurooncol..

[6] Connolly D.T., Heuvelman D.M., Nelson R., Olander J.V., Eppley B.L., Delfino J.J., Siegel N.R., Leimgruber R.M., Feder J. (1989). Tumor vascular permeability factor stimulates endothelial cell growth and angiogenesis. J. Clin. Investig..

[7] Nonoguchi N., Miyatake S.-I., Fukumoto M., Furuse M., Hiramatsu R., Kawabata S., Kuroiwa T., Tsuji M., Fukumoto M., Ono K. (2011). The distribution of vascular endothelial growth factor-producing cells in clinical radiation necrosis of the brain: Pathological consideration of their potential roles. J. Neurooncol..

[8] Yoshii Y. (2008). Pathological review of late cerebral radionecrosis. Brain Tumor. Pathol..

[9] Patel U., Patel A., Cobb C., Benkers T., Vermeulen S. (2014). The management of brain necrosis as a result of SRS treatment for intra-cranial tumors. Transl. Cancer Res..

[10] Boothe D., Young R., Yamada Y., Prager A., Chan T., Beal K. (2013). Bevacizumab as a treatment for radiation necrosis of brain metastases post stereotactic radiosurgery. Neuro Oncol..

[11] Glantz M.J., Burger P.C., Friedman A.H., Radtke R.A., Massey E.W., Schold S.C. (1994). Treatment of radiation-induced nervous system injury with heparin and warfarin. Neurology.

[12] Jacobson G., Bhatia S., Smith B.J., Button A.M., Bodeker K., Buatti J. (2013). Randomized trial of pentoxifylline and vitamin E vs standard follow-up after breast irradiation to prevent breast fibrosis, evaluated by tissue compliance meter. Int. J. Radiat. Oncol. Biol. Phys..

[13] Kaidar-Person O., Marks L.B., Jones E.L. (2018). Pentoxifylline and vitamin E for treatment or prevention of radiation-induced fibrosis in patients with breast cancer. Breast J..

[14] Williamson R., Kondziolka D., Kanaan H., Lunsford L.D., Flickinger J.C. (2008). Adverse radiation effects after radiosurgery may benefit from oral vitamin E and pentoxifylline therapy: A pilot study. Stereotact. Funct. Neurosurg..

[15] 3D Slicer Image Computing Platform. https://www.slicer.org/.

[16] Fedorov A., Beichel R., Kalpathy-Cramer J., Finet J., Fillion-Robin J.-C., Pujol S., Bauer C., Jennings D., Fennessy F., Sonka M. (2012). 3D Slicer as an image computing platform for the Quantitative Imaging Network. Magn. Reson. Imaging.

[17] Karlton W., Somayeh M. (2020). Time Frequency Analysis of Wavelet and Fourier Transform. Wavelet Theory.

[18] van Griethuysen J.J.M., Fedorov A., Parmar C., Hosny A., Aucoin N., Narayan V., Beets-Tan R.G.H., Fillion-Robin J.-C., Pieper S., Aerts H.J.W.L. (2017). Computational Radiomics System to Decode the Radiographic Phenotype. Cancer Res..

[19] Xanthopoulos P., Pardalos P.M., Trafalis T.B., Xanthopoulos P., Pardalos P.M., Trafalis T.B. (2013). Linear Discriminant Analysis. Robust Data Mining.

[20] Pedregosa F., Varoquaux G., Gramfort A., Michel V., Thirion B. (2011). Scikit-learn: Machine Learning in Python. J. Mach. Learn. Res..

[21] Cervantes J., Garcia-Lamont F., Rodríguez-Mazahua L., Lopez A. (2020). A comprehensive survey on support vector machine classification: Applications, challenges and trends. Neurocomputing.

[22] Chawla N.V., Bowyer K.W., Hall L.O., Kegelmeyer W.P. (2002). SMOTE: Synthetic minority over-sampling technique. J. Artif. Intell. Res..

[23] Zhang K., Abdoli N., Gilley P., Sadri Y., Chen X., Thai T.C., Dockery L., Moore K., Mannel R.S., Qiu Y. (2023). Developing a Novel Image Marker to Predict the Responses of Neoadjuvant Chemotherapy (NACT) for Ovarian Cancer Patients. arXiv.

[24] Bradley A.P. (1997). The use of the area under the ROC curve in the evaluation of machine learning algorithms. Pattern Recognit..

[25] Prabhu R.S., Press R.H., Patel K.R., Boselli D.M., Symanowski J.T., Lankford S.P., McCammon R.J., Moeller B.J., Heinzerling J.H., Fasola C.E. (2017). Single-Fraction Stereotactic Radiosurgery (SRS) Alone Versus Surgical Resection and SRS for Large Brain Metastases: A Multi-institutional Analysis. Int. J. Radiat. Oncol. Biol. Phys..

[26] Kerschbaumer J., Demetz M., Krigers A., Nevinny-Stickel M., Thomé C., Freyschlag C.F. (2021). Risk Factors for Radiation Necrosis in Patients Undergoing Cranial Stereotactic Radiosurgery. Cancers.

[27] Tanenbaum D.G., Buchwald Z.S., Jhaveri J., Schreibmann E., Switchenko J.M., Prabhu R.S., Chowdhary M., Abugideiri M., Pfister N.T., Eaton B. (2020). Dosimetric Factors Related to Radiation Necrosis After 5-Fraction Radiosurgery for Patients with Resected Brain Metastases. Pract. Radiat. Oncol..

[28] Acquitter C., Piram L., Sabatini U., Gilhodes J., Cohen-Jonathan E.M., Ken S., Lemasson B. (2022). Radiomics-Based Detection of Radionecrosis Using Harmonized Multiparametric MRI. Cancers.

[29] Peng L., Parekh V., Huang P., Lin D.D., Sheikh K., Baker B., Kirschbaum T., Silvestri F., Son J., Robinson A. (2018). Distinguishing True Progression From Radionecrosis After Stereotactic Radiation Therapy for Brain Metastases With Machine Learning and Radiomics. Int. J. Radiat. Oncol. Biol. Phys..

[30] Zhang Z., Yang J., Ho A., Jiang W., Logan J., Wang X., Brown P.D., McGovern S.L., Guha-Thakurta N., Ferguson S.D. (2018). A predictive model for distinguishing radiation necrosis from tumour progression after gamma knife radiosurgery based on radiomic features from MR images. Eur. Radiol..

[31] Khan M., Zhao Z., Arooj S., Liao G. (2021). Bevacizumab for radiation necrosis following radiotherapy of brain metastatic disease: A systematic review & meta-analysis. BMC Cancer.

[32] Zhuang H., Shi S., Yuan Z., Chang J.Y. (2019). Bevacizumab treatment for radiation brain necrosis: Mechanism, efficacy and issues. Mol. Cancer.

[33] Co J., De Moraes M.V., Katznelson R., Evans A.W., Shultz D., Laperriere N., Millar B.-A., Berlin A., Kongkham P., Tsang D.S. (2020). Hyperbaric Oxygen for Radiation Necrosis of the Brain. Can. J. Neurol. Sci..

[34] Smith J.A., Fenderson J.L. (2020). Diving Into Radiation Necrosis: Hyperbaric Oxygen Therapy in Cerebral Radiation Necrosis. JCO Oncol. Pract..

[35] Lefaix J.-L., Delanian S., Vozenin M.-C., Leplat J.-J., Tricaud Y., Martin M. (1999). Striking regression of subcutaneous fibrosis induced by high doses of gamma rays using a combination of pentoxifylline and alpha-tocopherol: An experimental study. Int. J. Radiat. Oncol. Biol. Phys..

[36] Delanian S., Chatel C., Porcher R., Depondt J., Lefaix J.-L. (2011). Complete restoration of refractory mandibular osteoradionecrosis by prolonged treatment with a pentoxifylline-tocopherol-clodronate combination (PENTOCLO): A phase II trial. Int. J. Radiat. Oncol. Biol. Phys..

[37] Delanian S., Depondt J., Lefaix J.L. (2005). Major healing of refractory mandible osteoradionecrosis after treatment combining pentoxifylline and tocopherol: A phase II trial. Head Neck.

[38] Andrés L., Vicente B., David M., Christakis C. (2016). Texture Analysis in Magnetic Resonance Imaging: Review and Considerations for Future Applications. Assessment of Cellular and Organ Function and Dysfunction Using Direct and Derived MRI Methodologies.

[39] Fink K.R., Fink J.R. (2013). Imaging of brain metastases. Surg. Neurol. Int..

[40] Demircioglu A. (2022). The effect of preprocessing filters on predictive performance in radiomics. Eur. Radiol. Exp..

[41] Salari E., Elsamaloty H., Ray A., Hadziahmetovic M., Parsai E.I. (2023). Differentiating Radiation Necrosis and Metastatic Progression in Brain Tumors Using Radiomics and Machine Learning. Am. J. Clin. Oncol..

