# Supplementary material for: Radiomic Analysis of Treatment Effect for Patients with Radiation Necrosis Treated with Pentoxifylline and Vitamin E
Source: Tomography. 2024 Sep 9;10(9):1501–12. doi: 10.3390/tomography10090110 (PMC11435669; doi:10.3390/tomography10090110)
Supplement: Supplementary file 1 [file tomography-10-00110-s001.zip › tomography-3041306-supplementary.pdf]

Supplementary

Pentoxifylline and Vitamin E for the Treatment of Radiation Necrosis: A Retrospective Cohort Analysis

Supplementary Table S1: Highest weighted radiomic features in SVM models of original and LLH-filtered images

	TOP TEN FEATURES: ORIGINAL (NO FILTERS)
**1**	original_glcm_Imc2
**2**	original_glrlm_ShortRunEmphasis
**3**	original_glcm_Idmn
**4**	original_shape_LeastAxisLength
**5**	original_firstorder_Median
**6**	original_glszm_SmallAreaLowGrayLevelEmphasis
**7**	original_ngtdm_Contrast
**8**	original_gldm_SmallDependenceLowGrayLevelEmphasis
**9**	original_gldm_LowGrayLevelEmphasis
**10**	original_shape_MinorAxisLength

	TOP TEN FEATURES: WAVELET FILTER (LLH)
**1**	glcm_Imc1
**2**	glcm_Correlation
**3**	ngtdm_Coarseness
**4**	glszm_ZonePercentage
**5**	glcm_Imc2
**6**	gldm_DependenceNonUniformity
**7**	gldm_SmallDependenceEmphasis
**8**	glcm_ClusterShade
**9**	glcm_MaximumProbability
**10**	irstorder_Minimum

Supplementary Table S2: Performance of radiomics models with varying wavelet filters with and without SMOTE

	AUC with SMOTE	AUC without SMOTE
Original (no filter)	0.47±0.030	0.50±0.023
HHH	0.46±0.119	0.48±0.106
HHL	0.59±0.111	0.57±0.111
HLH	0.57±0.114	0.53±0.116
HLL	0.51±0.107	0.51±0.109
LHH	0.50±0.099	0.47±0.097
LHL	0.63±0.097	0.60±0.098
LLH	0.69±0.091	0.60±0.067
LLL	0.45±0.055	0.46±0.041
